# Mouse intestinal tuft cells express advillin but not villin

**DOI:** 10.1038/s41598-020-65469-0

**Published:** 2020-06-01

**Authors:** Amin Esmaeilniakooshkghazi, Sudeep P. George, Ritwika Biswas, Seema Khurana

**Affiliations:** 1 Department of Biology and Biochemistry, University of Houston, Houston, Texas USA; 2 Department of Allied Health, Baylor College of Medicine, Houston, Texas USA

**Keywords:** Cytoskeleton, Predictive markers, Gastrointestinal diseases, Gastrointestinal models, Ileum

## Abstract

Tuft (or brush) cells are solitary chemosensory cells scattered throughout the epithelia of the respiratory and alimentary tract. The actin-binding protein villin (Vil1) is used as a marker of tuft cells and the villin promoter is frequently used to drive expression of the Cre recombinase in tuft cells. While there is widespread agreement about the expression of villin in tuft cells there are several disagreements related to tuft cell lineage commitment and function. We now show that many of these inconsistencies could be resolved by our surprising finding that intestinal tuft cells, in fact, do not express villin protein. Furthermore, we show that a related actin-binding protein, advillin which shares 75% homology with villin, has a tuft cell restricted expression in the gastrointestinal epithelium. Our study identifies advillin as a marker of tuft cells and provides a mechanism for driving gene expression in tuft cells but not in other epithelial cells of the gastrointestinal tract. Our findings fundamentally change the way we identify and study intestinal tuft cells.

## Introduction

Tuft (also called brush, multivesicular, fibrovesicular, caveolated) cells are unusual epithelial cells that have been identified in numerous epithelial tissue including the salivary glands, stomach, gall bladder, bile duct, pancreatic duct, small intestine, cecum, colon, nasal cavity, auditory tube, trachea, urethra, and the thymus of multiple species, all with remarkably similar ultrastructure^1,2^. Compared to other mucosal epithelial cells in the alimentary and respiratory tract, tuft cells contain numerous intermediate filaments, microtubules and a dense network of actin and actin-binding proteins suggesting a major role for the cytoskeleton in their morphology and function^3^. Additionally, unlike enterocytes, tuft cells are characterized by an apical candle-like “tuft” or “brush” of longer and thicker microvilli that extend into the perinuclear region and like enterocytes the tuft/brush is composed of actin filaments cross-linked by the actin bundling proteins villin-1 (henceforth referred to as villin) and fimbrin^4^. Consistent with that finding, villin was the first marker used to identify tuft/brush cells, despite the fact that villin is also expressed in differentiated epithelial cells of the intestine^5,6^. The villin promoter has also been used extensively to drive gene expression or gene deletion from tuft/brush cells. One consequence of that is that data obtained using the *Vil1-Cre* or *Vil1-Cre/ERT2* mice to study tuft cells, have been difficult to recapitulate when compared to similar studies done with other non-villin recombinase drivers such as the *Rosa26-Cre/ERT2*, doublecortin like kinase 1, *Dclk1-Cre*, or the leucine rich repeat containing G protein-coupled receptor 5, *Lgr5*^+^*-Cre*^7–12^. Similarly, the microtubule associated DCLK1, the transient receptor potential cation channel (TRPM5), prostaglandin-endoperoxide synthase 1 (PTGS1) are used to identify tuft cells, although all these proteins are also expressed in other cells within the gastrointestinal and respiratory tract and at levels comparable to those seen in tuft/brush cells^11,13–17^. Consequently, pairing these markers with each other, together with the unique candle-like “tufted” morphology are frequently employed to identify tuft/brush cells. Nonetheless, we note that within the gastrointestinal tissue, there is no definitive marker that is restricted to tuft cells. Consequently, the lineage of tuft cells as well as their functions have remained poorly understood largely because neither tuft cell deficient mice have been generated nor is it possible to modulate gene expression and activity only in intestinal tuft cells.

Advillin is a member of the villin/gelsolin superfamily that shares the highest structural homology with villin with a shared six domain structure and a carboxyl-terminal headpiece domain^18^. Interestingly, villin was identified in several chemosensory and mechanosensory cells that share many of the structural and functional characteristics of tuft cells. This includes Merkel cells in the skin, taste receptor cells, and exocrine glands with an endodermic lineage such as the thymus^19–24^. Villin was also frequently used as a marker for Merkel cells and taste receptor cells. However, more recently using immunohistochemistry and two advillin reporter mouse strains it has been shown that Merkel cells express advillin; while single cell RNA-Seq studies have demonstrated that Merkel cells contain advillin not villin mRNA^20,25^. Similarly, advillin protein has been identified in the taste receptor cells and advillin not villin mRNA has been identified using single-cell RNA-Seq on taste bud cells; likewise thymic brush cells have also been shown to express high levels of advillin but not villin mRNA^26–28^. More importantly, one of these studies has also identified advillin protein in solitary enteroendocrine-like epithelial cells in the mouse duodenal epithelium^20^. Although, we note that no markers of enteroendocrine cells were used in this study and that these cells appear to have the unique morphology of candle-like “tufted” cells rather than enteroendocrine cells^20^. In light of these discoveries, we reasoned that a careful evaluation of the villin family protein expression in intestinal tuft cells (and by extension in brush cells) was necessitated. Here, we report our surprising finding that intestinal tuft cells, in fact, do not express villin protein and that like other chemosensory and mechanosensory cells described above, they express advillin protein. To our surprise, a careful evaluation of more recently published studies that have employed single cell RNA-Seq to identify genes expressed in tuft/brush cells also identify advillin mRNA but do not describe villin mRNA in tuft/brush cells^9,29–33^. However, none of these studies describe the significance of these data. In light of our findings, we suggest that a thorough assessment of tuft cell lineage commitment and function is warranted. Furthermore, while it has been suggested that like trigeminal ganglia neurons, enteric neurons may also express advillin, immunohistochemistry and RNA-Seq studies reveal the absence of advillin from enteric neurons or enteric glia cells^20,34,35^. Based on that we propose that advillin expression in the gastrointestinal tract is restricted to tuft cells.

## Results and Discussion

**Figure 1:**
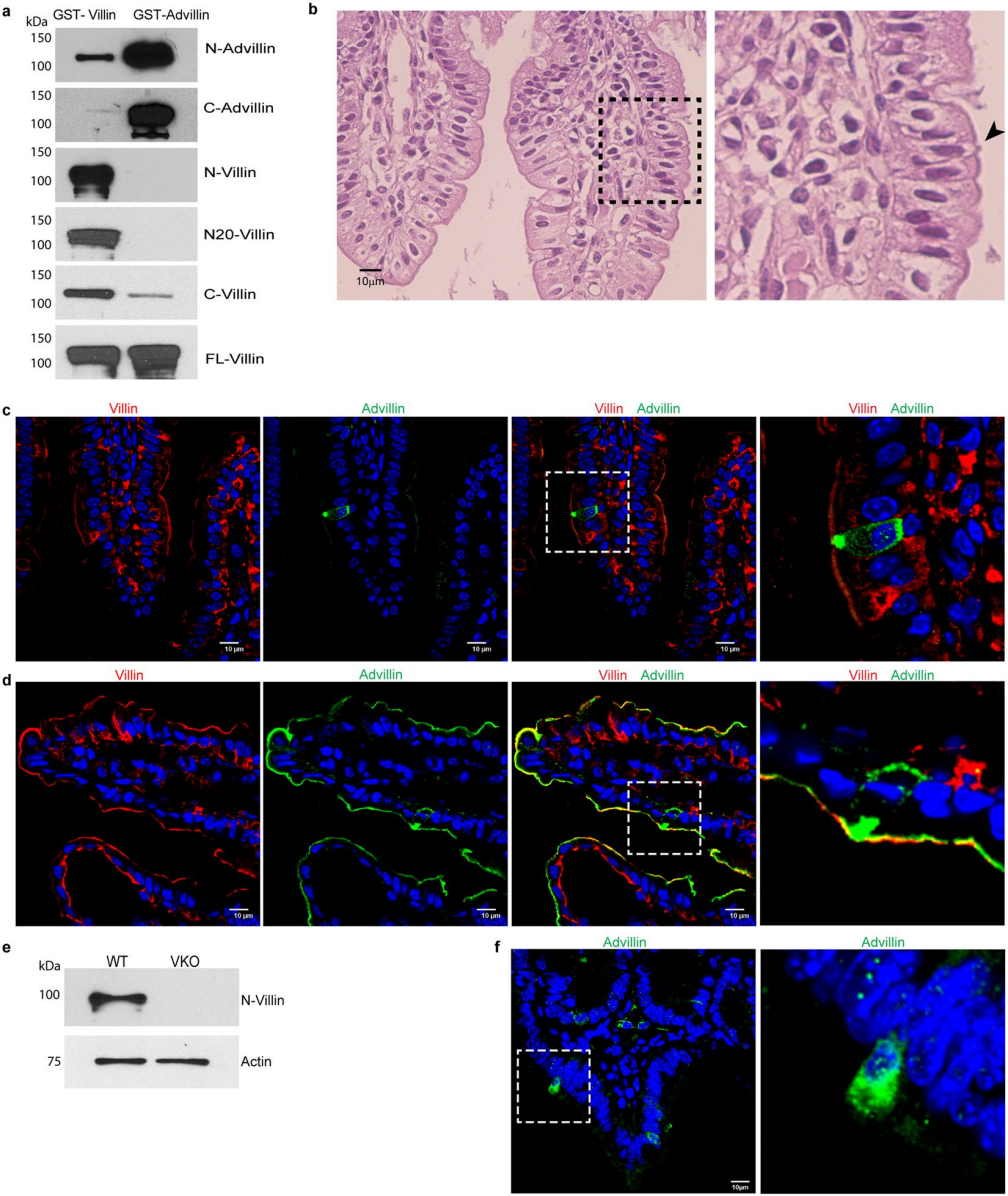
Mouse intestinal cells that express advillin do not express villin. (**a**) Specificity of villin and advillin antibodies was confirmed by Western analysis using recombinant full-length human villin and human advillin proteins. Two antibodies raised against the amino-terminus of villin (N-villin and N20-villin) did not cross-react with advillin compared to antibodies raised against the carboxyl-terminus of villin (C-villin) or full-length villin (FL-villin) protein. Antibodies raised against the amino-terminus of advillin (N-advillin) and those raised against the carboxyl-terminus of advillin (C-advillin) detect advillin better than they cross-react with villin. Original full length data are available in supplementary Fig S1. (**b**) H & E staining of mouse distal ileum identifies solitary cells with candle-like “tufted morphology” (identified by arrowhead). Right panel shows higher magnification of the boxed area. (**c**) Immunohistochemistry of paraffin embedded tissue from distal ileum of C57BL/6 J mice using N-villin (red) and advillin (green) antibodies. Nuclei are counter stained with DAPI. N-villin antibody does not cross-react with advillin in cells that express advillin in apical tufts and along the basolateral surface. Right panel shows higher magnification of boxed area. (**d**) Immunohistochemistry of cryopreserved tissue from distal ileum of C57BL/6 J mice using N-villin (red) and advillin (green) antibodies. Nuclei are counter stained with DAPI. N-villin does not co-localize with advillin in cells that express advillin in apical tufts and along the basolateral surface. Right panel shows higher magnification of boxed area. (**e**) Western analysis of distal ileal tissue from villin knockout (VKO) mice and their wild type (WT) littermates show the absence of villin protein in VKO mice. Original full length data are available in supplementary Fig S2. (**f**) Immunohistochemistry of paraffin embedded tissue from distal ileum of villin knockout mice identify advillin but not villin expressing cells. Nuclei are counter stained with DAPI. Right panel shows higher magnification of boxed area. Data shown in Western blots are representative of three independent experiments and in immunohistochemistry of *n* = 5 animals. Scale bars represent 10 μm.

**Figure 2:**
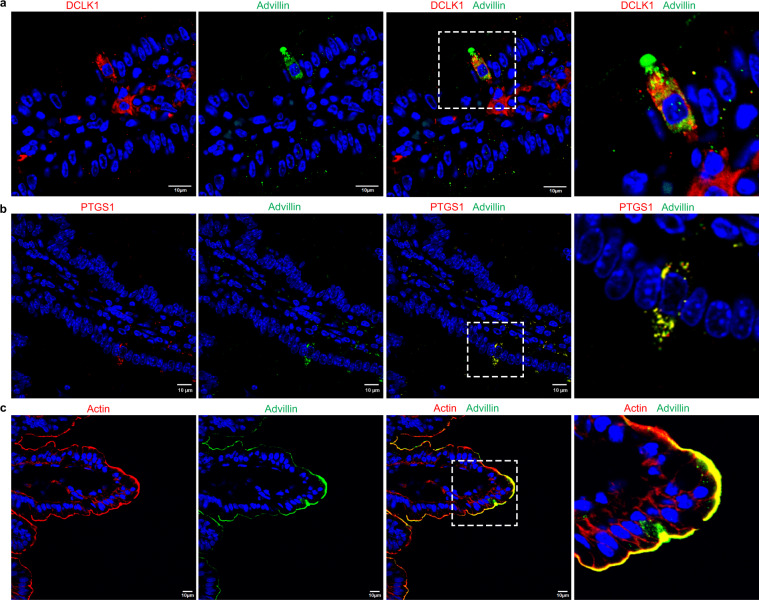
Intestinal tuft cells express advillin. (**a**) Immunohistochemistry of paraffin-embedded tissue from distal ileum of C57BL/6 J mice using advillin (green) and DCLK1 (red) antibodies. Nuclei are counter stained with DAPI. Both proteins co-localize in the same cell. Note that not all DCLK1 positive cells are positive for advillin expression. Right panel shows higher magnification of boxed area and shows co-localization of DCLK1 and advillin in a tuft cell. (**b**) Immunohistochemistry of paraffin embedded tissue from distal ileum of C57BL/6 J mice using advillin (green) and PTGS1 (red) antibodies. Nuclei are counter stained with DAPI. Right panel shows higher magnification of boxed area and co-localization of PTGS1 and advillin in a tuft cell. (**c**) Immunohistochemistry of cryopreserved tissue from distal ileum of C57BL/6 J mice using Alexa Fluor 568 Phalloidin (red) and advillin (green). Nuclei are counter stained with DAPI. Right panel shows higher magnification of boxed area and co-localization of advillin and F-actin at the apical surface of a tuft cell. Data shown are representative of *n* = 5 animals. Scale bar represents 10 μm.

Based on a recent report, we noted that both villin and advillin expressing cells are present in the gastrointestinal epithelium^20^. Since villin and advillin share very significant (~75%) structural homology, our goal was to first identify antibodies that can distinguish between these two proteins^18^. Using recombinant human villin and advillin proteins we determined that most villin and advillin antibodies cross-react (Fig. 1a). We have previously shown that these antibodies do not cross-react with GST^36,37^. Careful characterization allowed us to identify two antibodies raised against the amino-terminus of villin (N-Villin and N20-Villin) that do not cross react with advillin (Fig. 1a). The region of human villin used to generate these antibodies shows some divergence when compared to human advillin gene. Furthermore, we noted that multiple advillin antibodies identify advillin much better than they cross-react with villin, this includes an advillin antibody raised against its amino-terminus (a.a. 440-526; N-advillin) and one raised against the carboxyl-terminus of advillin (a.a. 750-813; C-advillin). Using H & E (Hematoxylin and Eosin) staining of mouse distal ileum we identified less than 0.5% of the cells with a unique candle-like “tufted” morphology associated with intestinal tuft cells (Fig. 1b). Consistent with previous reports, these cells were determined to be approximately 10 μm long and approximately 5 μm in thickness. Based on the thickness of these cells, and the paucity of these cells in the normal mouse intestine, several attempts to use paraffin-embedded serial sections to correlate the morphological features of the same tuft cells with the expression of villin, advillin, and other known tuft cell markers, were unsuccessful. This may also be, one of the reasons, why such studies have not been performed previously. With that knowledge, we identified advillin expressing cells in the terminal ileum of C57/BL6 mice that resembled histomorphologically tuft cells, rather than enteroendocrine cells with a candle-like “tufted” morphology (Fig. 1c)^20^. Using paraffin-embedded ileal tissue, we noted that cells with this tufted morphology do not express villin protein (Fig. 1c). Cryopreserved tissue sections (which preserve F-actin better than paraffin embedded tissue) from distal ileum also showed solitary tuft cell-like advillin expressing cells that do not express villin protein (Fig. 1d). The advillin expressing cells show protein localization at the apical tufts and at the basolateral surface in what appears to be vesicular structures (Fig. 1c,d). It may be noted that previous studies have identified villin in the apical tufts and along the basolateral membrane of tuft cells^38^. This is different from the apical brush border restricted distribution of villin in enterocytes. Using the villin knockout (VKO) mice we confirmed the absence of villin (as shown here and as reported previously; Fig. 1e) and the presence of advillin expressing cells that resembled morphologically, tuft cells (Fig. 1f)^39^. Please note the absence of advillin staining in the gastrointestinal epithelium of the VKO mice, demonstrating that in the absence of villin protein the advillin antibodies do not detect any other cells in the epithelium (Fig. 1f). Tuft cells are marked by DCLK1 and are enriched in PTGS1^9,40^. We confirmed our hypothesis that these advillin expressing cells are tuft cells by double labeling with antibodies against these commonly used markers of tuft/brush cells (Fig. 2a,b). Not all DCLK1 positive cells were positive for advillin and likely represent insulinoma-associated 1 positive enteroendocrine cells as has been suggested before^11,13^. Cryopreserved tissue sections show F-actin enriched in candle-like apical “tufts” of epithelial cells that are also positive for advillin expression (Fig. 2c). As described before, a unique morphological feature of tuft cells is the presence of axial bundles of actin filaments with a candle-like tuft or brush morphology^41^. This unique morphology identifies tuft cells and we find advillin but not villin enriched in these cells of the intestinal epithelium.

**Figure 3:**
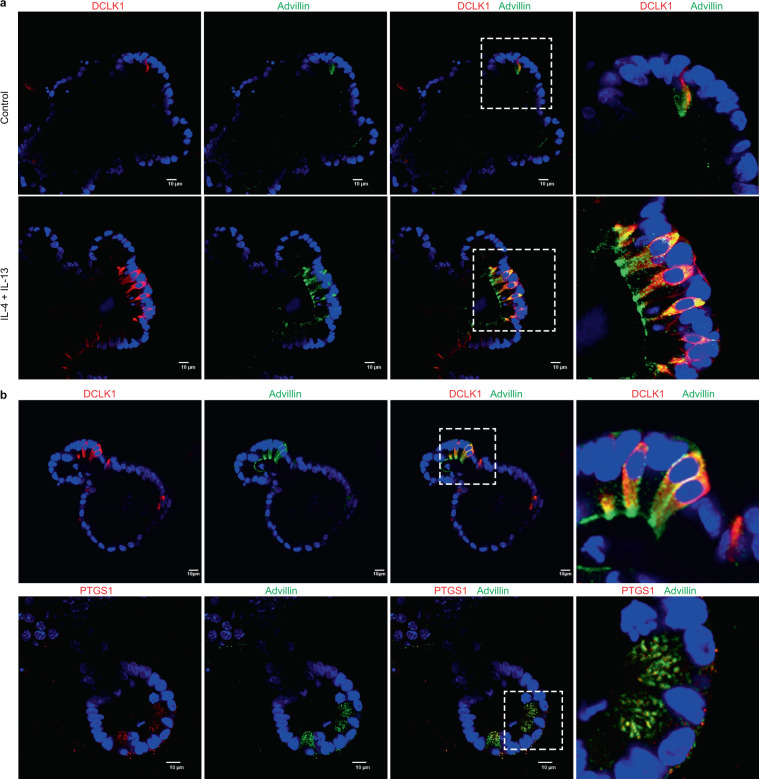
In intestinal enteroids, tuft cells express advillin. (**a**) Immuno-histochemistry of enteroids from distal ileum of C57BL/6 J mice shows tuft cell hyperplasia 72 hours post IL-4 and IL-13 treatment. Control refers to untreated enteroids from C57BL/6 J mice. Advillin (green) and DCLK1 (red) co-localization was used to identify tuft cells. Nuclei are counter stained with DAPI. Right panel shows higher magnification of the boxed area. (**b**) Immunohistochemistry of IL-4 and IL-13 treated enteroids from distal ileum of C57BL/6 J mice, show co-localization in tuft cells of advillin (green) and DCLK1 (red) in the upper panel; and advillin and PTGS1 (red) in the lower panel. Nuclei were counter stained with DAPI. Right panels show higher magnification of boxed area. Data shown are representative of *n* = 5 animals. Scale bar represents 10 μm.

**Figure 4:**
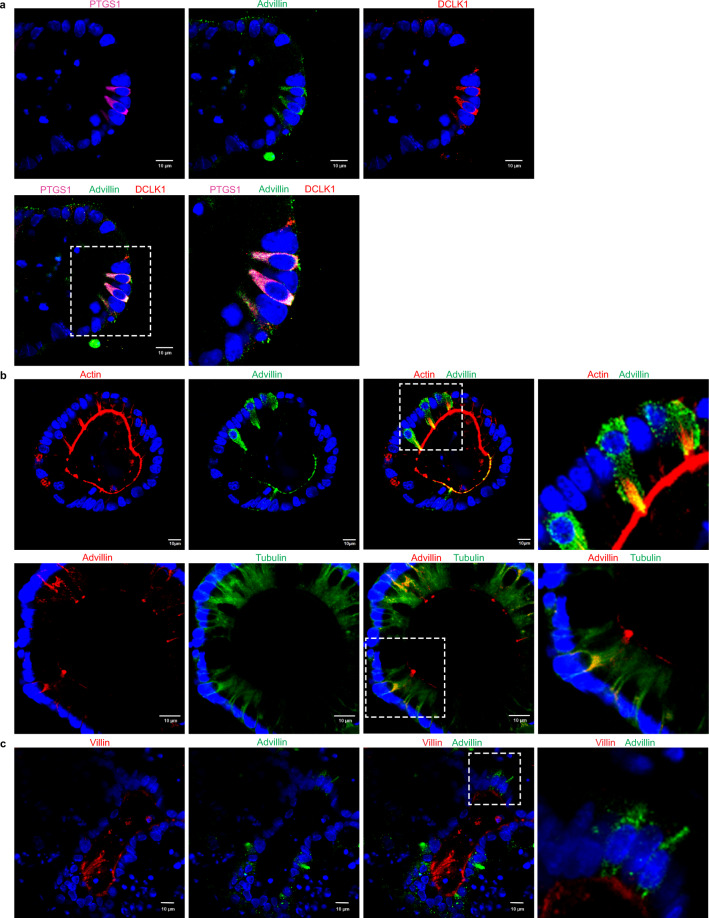
Tuft cells positive for advillin are negative for villin. (**a**) Immunohistochemistry and Virtual Channel confocal microscopy of IL-4 and IL-13 treated enteroids from distal ileum of C57BL/6 J mice, show co-localization of advillin (green), DCLK1 (red) and PTGS1 (magenta) within the same cells identified as tuft cells. Nuclei are counter stained with DAPI. Right panel shows higher magnification of boxed area. (**b**) Immunohistochemistry of IL-4 and IL-13 treated enteroids from distal ileum of C57BL/6 J mice show co-localization in tuft cells of advillin (green) with F-actin (red) in the upper panel; and co-localization of advillin (red) and tubulin (green) in the lower panel. Nuclei were counter stained with DAPI. Right panel shows higher magnification of boxed area. Note the co-localization of advillin to the apical tuft that terminates near the perinuclear region of the cells. Advillin appears in vesiculated structures that co-localize with tubulin near the lateral cell surface and in the cytoplasm. (**c**) Immunohistochemistry of IL-4 and IL-13 treated enteroids from distal ileum of C57BL/6 J mice show the absence of villin (red) from advillin (green) expressing tuft cells. Nuclei are counter stained with DAPI. Right panel shows higher magnification of boxed area. Data shown are representative of *n* = 5 animals. Scale bar represents 10 μm.

In the normal mouse intestine the number of tuft cells is very low (~0.5%) although this number can be increased 10-fold in the gut following parasitic (enteric metazoan and protozoan) colonization and infection^42,43^. Alternatively, this can be achieved by exogenous addition of interleukins (IL) IL-4 and IL-13 to isolated mouse enteroids, as reported before and as shown here (Fig. 3a)^43^. Similar to the intestinal tissue, *ex vivo* in the enteroids, advillin labeled the tuft cells as noted by co-localization of DCLK1 and PTGS1 (Fig. 3b). Using Virtual Channels to acquire multichannel confocal images, we show that all three proteins, PTGS1, DCLK1 and advillin are localized to the same cells (Fig. 4a). As expected, in mouse enteroids advillin co-localizes with the cytoskeletal proteins F-actin and tubulin (Fig. 4b). F-actin and advillin localized primarily to the eponymous apical tuft consisting of actin microfilaments that terminate at the perinuclear region. In contrast, tubulin localizes to the upper half of the cell and the basolateral surface of tuft cells where advillin co-localizes with the cytoplasmic tubulin. As reported before and as shown here, tubulin expression in the upper half of the cell is also unique to tuft cells and is never seen in other gastrointestinal or respiratory epithelial cells^3^. Similar to data shown in Figs. 1c,d,f, 2a–c, 3a,b, the expression of advillin in tuft cells appears to be associated with vesicular structures (Fig. 4b,c). More notably, in mouse enteroids like in the mouse intestine, advillin expressing cells do not express villin protein (Fig. 4c).

**Figure 5:**
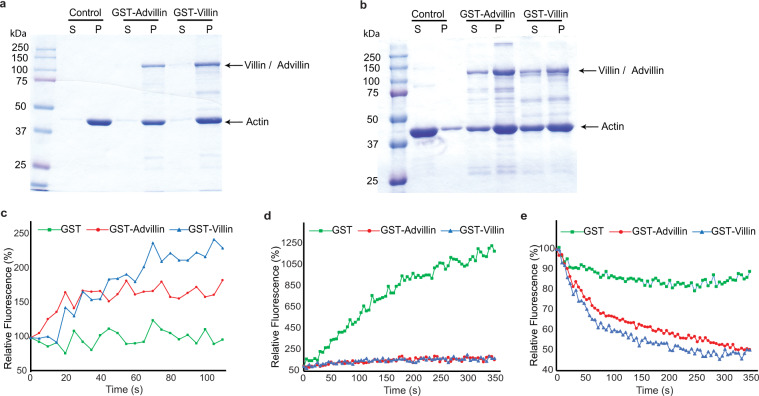
Regulation of actin dynamics by villin and advillin. (**a**) Villin and advillin bind F-actin. Recombinant villin and advillin proteins (60 nM) were incubated with 3 μM F-actin and centrifuged at 200,000 x g for 15 min. The supernatant (S) precipitated with 2 volumes of acetone and the pellet (P) were analyzed by SDS-PAGE and gels were stained with GelCode Blue. Control refers to assay in the absence of either protein. (**b**) Villin and advillin bundle F-actin. Recombinant villin and advillin proteins (1 μM) were incubated with F-actin and centrifuged at 10,000 x g for 15 min. The supernatant (S) and pellet (P) fractions were analyzed by SDS-PAGE and gels were stained with GelCode Blue. Control refers to assay in the absence of either protein. (**c**) Villin and advillin (60 nM each) nucleate actin filaments. Pyrene-labeled G-actin (6 μM) was polymerized in the presence of villin or advillin protein and fluorescence intensity measured over time. (**d**) Villin and advillin (60 nM each) cap actin filaments. Polymerization of 1.4 μM pyrene-labeled G-actin in the presence of F-actin seeds (290 nM) was measured in the presence of villin or advillin protein. (**e**) Villin and advillin sever actin filaments. Pyrene-labeled F-actin (1 μM) was diluted to 0.1 μM in actin-polymerizing buffer in the presence of villin or advillin protein (60 nM each) and the decrease in fluorescence intensity was followed over time. All gels are representative of three experiments with similar results. In all actin kinetic assays, values represent the mean of three independent experiments.

Although advillin was identified over two decades ago as a new member of the gelsolin/villin family of proteins, the advillin protein has not been studied to determine either its actin binding ability or its ability to modify actin dynamics^18^. Based on that we elected to study, for the first time, the actin regulatory functions of advillin. All *in vitro* studies were performed with comparable levels of recombinant villin and advillin proteins. Using a standard *in vitro* assay for the measurement of actin binding by purified recombinant villin and advillin, we show that like villin, advillin is an actin binding protein (Fig. 5a)^44^. Advillin contains a villin-like carboxyl-terminal headpiece domain that is associated with villin’s bundling function^18^. Using a standard sedimentation assay for actin bundling we now show that this domain in advillin is functional and that advillin bundles actin similar to the villin protein (Fig. 5b). Advillin also shares the six domain structure of gelsolin and villin that is responsible for the actin depolymerizing functions of both proteins^6^. We now show, for the first time, that like its family members villin and gelsolin, advillin can nucleate, cap and sever actin filaments (Fig. 5c–e). These data demonstrate, for the first time, that advillin shares structural but also functional homology with other members of its family. Both villin and advillin are expressed in the gastrointestinal epithelium but are restricted to distinct cell types. While villin expression is restricted to differentiated intestinal epithelial cells, advillin expression is restricted to the chemosensory tuft cells. This lack of villin from tuft cells may also explain the unique ultrastructural features of tuft cells not shared by enterocytes namely, an apical tuft of stiff microvilli with long microvillar actin rootlets and no terminal web^45^. Furthermore, we hypothesize that unlike the restricted apical brush border localization of villin, the apical and basolateral expression of advillin in tuft cells may also have functions unique to solitary sensory cells. Additionally, these findings suggest that enterocytes and tuft cells may also have distinct lineage.

Since the development of the *villin-Cre* and *villin-Cre/ERT2* mouse lines, more than a dozen studies have relied on them for labeling or knocking out genes in the intestinal tuft cells. While these studies do not claim gene expression or gene deletion restricted to tuft cells, they all identify villin expression in the intestinal tuft cells, and villin expression in tuft cells was important for the interpretations related to the lineage and function of tuft cells. For instance, two independent groups used the atonal bHLH transcription factor 1, *Atoh1* ^*flox/flox*^ mice to induce *Atoh1* deletion from the intestinal epithelium. One group crossed these mice with the *Vil1-Cre/ERT2* mice and reported the complete loss of tuft cells^46^. Two other groups crossed the *Atoh1*^*flox/flox*^ mice to *Rosa26-Cre/ERT2* mice and reported the opposite namely, that tuft cells are preserved or that there is a significant increase in the number of tuft cells^11,12^. However, a subsequent study using the *Lgr5-Cre/ERT2* mice crossed to *Atoh1*^*flox/flox*^ mice also demonstrated that loss of *Atoh1* from Lgr5^+^ cells (which includes tuft cells) results in an increase in the number of tuft cells in the intestinal epithelium^12^. Despite that confirmation, the role of Atoh1 in tuft cell development remains controversial, leading some to suggest an Atoh1-dependent and Atoh1-independent lineage for intestinal tuft cells^47^. Similarly, a role for the regulatory associated protein of MTOR complex 1 (RPTOR) in tuft cell lineage has been reported using the *Vil1-Cre/ERT2* and the *Raptor*^*flox/flox*^ mice even though *ex vivo* enteroids derived from these mice have normal tuft cells^48^. The role of the Wnt target gene *Sox9* is likewise debatable. While several groups have demonstrated that DCLK1^+^ tuft cells express *Sox9* the *Vil1-Cre Sox9*^*flox/flox*^ mice show no effect of *Sox9* loss on tuft cell distribution^46,49,50^. The role of tuft cells in the regulation of intestinal repair is also discordant. Deleting *Dclk1* using the *Vil1*-*Cre Dclk1*^*flox/flox*^ model showed that animals fail to recover normal crypt-villus architecture following genotoxic insult or treatment with dextran sodium sulfate, suggesting a loss of epithelial regeneration accompanied by a dramatic loss of self-renewal pathways regulated by Notch and mammalian target of rapamycin (mTOR)^51,52^. Consistent with that, two independent studies using the *Vil1-Cre Dclk1*^*flox/flox*^ model demonstrated that tuft cells regulate DNA damage response and that the crypts of these mice have significantly higher number of apoptotic cells following radiation-induced injury^51,53^. In contrast, McKinley *et al*. demonstrated that when compared to most intestinal epithelial cells, tuft cells are extremely resistant to mucosal atrophy in response to acute fasting^33^. Additionally, using *Dclk1-Cre/ERT2 Rosa26-LacZ* reporter mice it was shown that tuft cells do not function as reserve stem cells further supporting the idea that tuft cells play no role in epithelial regeneration, a finding that was supported by studies done by Nakanishi *et al*.^12,54^ Our study underscores that the most likely explanation for many of these and similar conflicting findings related to tuft cell lineage commitment and function namely, the absence of villin protein from tuft cells. While the *villin-Cre* and *villin-Cre/ERT2* mice are used to target gene expression or gene loss from a limited number of adult gastrointestinal epithelial cells that endogenously express villin, namely the enterocytes, unexpected expression of *Cre* recombinase in cells that do not endogenously express villin such as goblet cells is known. This 'ectopic' expression of *villin-Cre* in goblet cells and potentially tuft cells should be approached with caution. It is also known that a given *Cre*-expressing mouse can have different recombination efficiencies for different floxed genes in different cell types. Our findings underscore the importance of testing the *Cre* expression outside the expected cell type and highlight the need for a greater degree of caution that must be used when empolying the *Vil1-Cre* or *villin-Cre/ERT2* mice to study gastrointestinal tuft cells. 

Bezencon *et al*. used the Trpm5 promoter to express enhanced green fluorescent protein (EGFP) in intestinal tuft cells followed by RNA-Seq of the isolated EGFP expressing cells^29^. Their study reported very high expression of advillin but not villin mRNA in tuft cells and they identified no advillin mRNA in other EGFP null cells of the gastrointestinal epithelium^29^. Based on that the authors even suggested that it was possible that previously published immunostaining of brush cells with villin antibodies resulted from cross-reactivity with advillin^29^. We note that multiple single-cell RNA-Seq studies published after that have also identified advillin but none have identified villin mRNA in tuft/brush cells and a few have also shown the absence of advillin mRNA from other intestinal epithelial cells including enterocytes^9,30–32^. Nevertheless, we also note that none of these studies address these discrepancies related to villin/advillin expression in tuft/brush cells. Using the advillin promoter driven EGFP expression and a Human Protein Atlas advillin antibody, Hunter and colleagues reported that advillin is expressed in the duodenum in solitary endocrine cells even though these cells appear to morphologically resemble the candle-like “tufted” cells^20^. Their study also did not include any specific marker of enteroendocrine cells. Additionally, the authors reported advillin expression in enteric neurons^20^. We note that while Hunter *et al*. identify cells in the Meissner’s plexus that are enriched in EGFP-advillin, these cells do not appear to be the same as those identified by the advillin antibody. More significantly, the same antibody does not identify any enteric neurons in the duodenum or any other sections of the gastrointestinal tract but identifies solitary cells in the gastrointestinal epithelium with a candle-like “tufted” morphology (https://www.proteinatlas.org/ENSG00000 135407-AVIL/tissue). Our own studies with these antibodies (N-advillin) identify advillin positive tuft cells. It is known that tuft cells communicate with afferent nerves and that PGP9.5-positive nerves make direct contact with duodenal tuft cells^29^. We suggest that one possibility is that Hunter *et al*. have identified enteric neurons (identified by EGFP-advillin) in close proximity of advillin expressing tuft cells^20^. Alternatively, this discrepancy could be attributed to the ectopic expression of EGFP advillin in enteric neurons.  There are a few single cell RNA-Seq studies on enteric neurons that do not identify advillin mRNA in enteric neurons or glia cells (Public databases: PanglaoDB; Single Cell Expression Atlas – EMBL-EBI)^34,35,55,56^, Based on that we suggest that advillin expression in the gastrointestinal tract may be restricted to the tuft cells. It is generally agreed that only tuft cell specific deletion of genes will provide the most definitive understanding of tuft cell lineage and function *in vivo*^7,31^. Unlike the *Vil1-Cre* or *Vil1-Cre/ERT2* mice the availability of the advillin Cre mouse allows, for the first time, the targeting of this subpopulation of gastrointestinal epithelial cells, rather than all epithelial cells. Such approaches are more likely to provide a detailed investigation of the molecular mechanisms and functions of intestinal tuft cells.

The exact function of advillin has not been investigated although its distribution to intestinal tuft cells (and by extension respiratory brush cells) indicates a role in chemosensing and initiation of immune type 2 response. The fact that tuft cells express advillin and not villin suggests that despite the significant structural and functional homology, advillin may be uniquely adapted to regulate chemosensory and mechanosensory functions of tuft cells. Advillin also differs from villin in significant ways such as advillin interacts with the scavenger receptor SREC-1 and contributes to neurite outgrowth *in vitro*^57–59^. Since tuft cells are found in contact with nerve fibers, advillin may have a unique function in regulating the enteric nervous system. That would also suggest that advillin plays a role in transferring sensory signals to the enteric nervous system.

## Methods

### Reagents

Murine recombinant epidermal growth factor, Advanced DMEM/F12, penicillin-streptomycin, HEPES buffer, Dulbecco’s Phosphate Buffered Saline and L-glutamine were bought from Gibco. Matrigel was from Corning, Inc. Noggin-conditioned medium and R-spondin conditioned medium (Noggin-producing cells and R-spondin-producing cells) were kindly provided by the Texas Medical Center Digestive Diseases Center (DDC) core. Acrylamide, SDS, Ammonium Persulfate, Tetramethylethylenediamine, N2 supplement, B27 supplement, Tissue-Tek**®** Optimal Cutting Temperature™ (O.C.T.) compound, and 24 well cell culture treated plate were from Thermo-Fisher Scientific. Glass bottom dishes were from MatTek.

### Antibodies

The following antibodies were used in the study: PTGS1 (NB100-867) from Novus Biologicals; N-advillin raised against the amino-terminus of advillin (a.a. 440-526), used in Human Protein Atlas is available from Sigma (HPA058864) and from Novus Biologicals (NBP2-34118); C-advillin antibody raised against the carboxyl-terminus of advillin (a.a. 750-813), from Abcam (ab72210); DCLK1 (H00009201-M03) from Abnova; Tubulin (ab131205) and N-Villin (ab201989) from Abcam; N20-Villin (sc33347), C-Villin (sc7672) from Santa Cruz Biotechnology, Inc.; FL-Villin (610359) from BD Transduction Laboratories; Hematoxylin and Eosin staining kit from VWR (470302-740); Alexa Fluor 568 phalloidin, Alexa Fluor 488, Alexa Fluor 647 and Alexa Fluor 555 from Thermo-Fisher Scientific. N-advillin from Novus Biologicals (NBP2-34118) was used in all immunohistochemical studies. Anti-fade mounting medium with DAPI was from Vector Laboratory. Actin Polymerization Biochem Kit was purchased from Cytoskeleton Inc. Recombinant murine IL-4 and IL-13 were purchased from Peprotech.

### Mice

All experimental protocols were approved by Institutional Animal Care and Use Committee (IACUC) of the University of Houston. Villin-1 knock-out (VKO) mice and their wild-type (WT) littermates have been described by us previously^60^. C57BL/6 J mice were purchased from the Jackson laboratory.

### Enteroid 3D culture and cytokines treatment

All methods were performed in accordance with the relevant guidelines and regulations. Crypt isolation and enteroid 3D cultures were derived from distal ileum of mice as previously described^61^. Organoid lines were passaged up to 10 times before experiments to ensure pure epithelial cultures. Enteroid 3D cultures were treated with a mixture of recombinant murine IL-4 (400 ng ml^−1^) and recombinant murine IL-13 (400 ng ml^−1^)^43^. Organoids were fixed and permeabilized for 1 hour using 3.7% paraformaldehyde and 0.2% Triton X-100. All images were acquired on Olympus Fluoview FV1200 Laser Scanning confocal microscope with a 60X NA 1.35 objective. The FV1200 Confocal microscope has three photodetectors, to image four fluorophores a Virtual Channel was created and imaging was performed under sequential mode.

### Immunohistochemistry using paraffin and Optimal Cutting Temperature (O.C.T.) embedded tissue

Tissue from distal ileum of mice were fixed and paraffin-embedded as described previously^61^. Hematoxylin and eosin (H & E) staining was performed as described previously^62^. To preserve the F-actin, some tissues were embedded in O.C.T. compound and stored at -80 °C. O.C.T. frozen blocks were sectioned 5 μm thick using Leica CM3050 Cryostat. The immunofluorescence studies were performed as described previously^61^. All fixed tissues were embedded and sectioned by the Texas Medical Center Digestive Diseases Center (DDC) core.

### Measurement of actin dynamics regulated by villin and advillin proteins

Recombinant full-length human villin and advillin proteins were cloned, expressed and purified as described previously^36,63^. Actin capping, nucleating and severing assays were performed as described by us^64^. Actin binding and actin bundling assays were performed as described by us previously^65^. Fluorescence measurements were performed using the FluoroMax-4 spectrofluorometer (Horiba Scientific).

## Supplementary information

Supplementary Data.
