# Supplementary material for: Mouse intestinal tuft cells express advillin but not villin
Source: Sci Rep. 2020 Jun 1;10:8877. doi: 10.1038/s41598-020-65469-0 (PMC7264147; doi:10.1038/s41598-020-65469-0)

Supplementary Data

Mouse intestinal tuft cells express advillin but not villin.

Amin Esmaeilniakooshkghazi, Sudeep P. George, Ritwika Biswas and Seema Khurana.

Figure S1. Western analysis using recombinant human villin and advillin proteins (full-length data for Figure 1a).

a, N-Advillin antibody has higher specificity for advillin than villin. **b,** C-Advillin antibody has higher specificity for advillin than villin. **c,** N-Villin antibody recognizes villin and not advillin. **d,** N20-Villin antibody recognizes villin and not advillin. **e,** C-Villin antibody recognizes both villin and advillin. **f,** Full-length villin antibody recognizes both villin and advillin.

Figure S2. Western analysis of distal ileal tissue from VKO mice show the absence of villin (full-length data for Figure 1e).

a, N-villin antibody shows the absence of villin in VKO mice. **b,** Actin antibody used as a positive control.

Figure S1

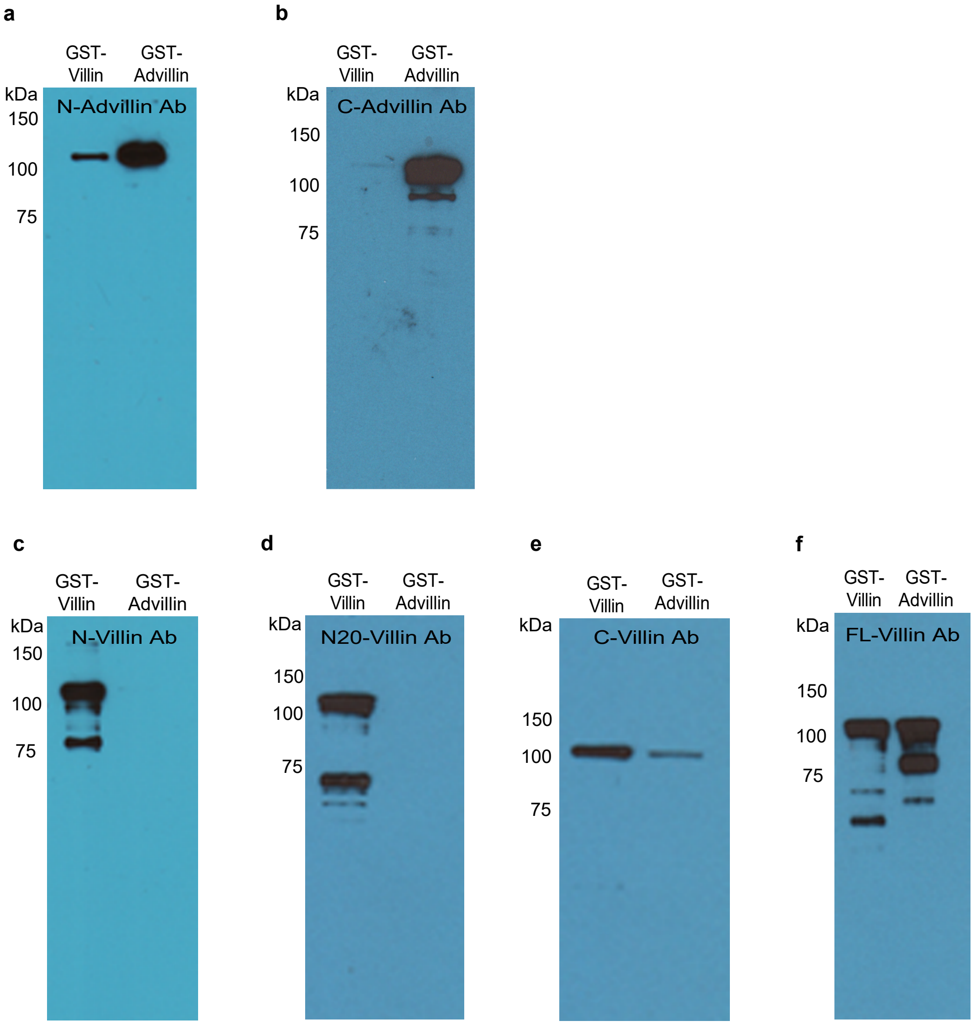

a
GST-Villin
GST-Advillin
kDa
150
100
75
N-Advillin Ab
b
GST-Villin
GST-Advillin
kDa
150
100
75
C-Advillin Ab
c
GST-Villin
GST-Advillin
kDa
150
100
75
N-Villin Ab
d
GST-Villin
GST-Advillin
kDa
150
100
75
N20-Villin Ab
e
GST-Villin
GST-Advillin
kDa
150
100
75
C-Villin Ab
f
GST-Villin
GST-Advillin
kDa
150
100
75
FL-Villin Ab

Figure S2

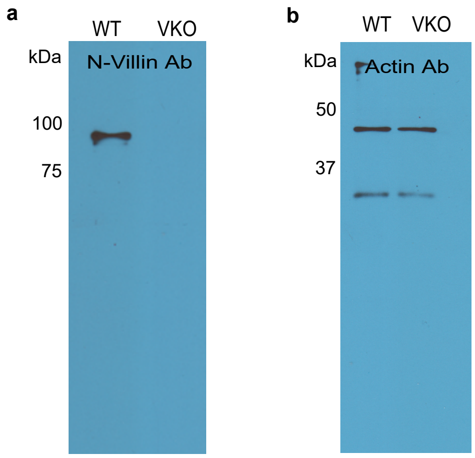

Supplement: Supplementary file 1 — Supplementary Data. [file 41598_2020_65469_MOESM1_ESM.pdf]
